# Iron deficiency anemia-related mortality trends in US older subjects, 1999 to 2019

**DOI:** 10.1007/s40520-025-02982-0

**Published:** 2025-03-22

**Authors:** Marco Zuin, Luigi Ferrucci, Giovanni Zuliani

**Affiliations:** 1 Department of Translational Medicine, University of Ferrara - UNIFE, 44124 Ferrara, Italy; 2 Translational Gerontology Branch, National Institute On Aging, National Institutes of Health, Baltimore, MD USA

**Keywords:** Iron deficiency anemia, Mortality, Gender, Race, Mortality trends

## Abstract

**Background:**

Previous investigations showed that the prevalence of iron deficiency is increasing in United States (US). However, data regarding iron deficiency anemia-related mortality trends are lacking. We assess the trends in iron deficiency anemia-related mortality in US adults aged 65 years or older over the last two decades.

**Methods:**

Iron-deficiency anemia-related deaths were ascertained using ICD-10 codes in the Centers for Disease Control and Prevention Wide-Ranging Online Data for Epidemiologic Research (CDC WONDER) database from 1999 to 2019. Age-adjusted mortality rates (AAMRs) were assessed using the Joinpoint regression modelling and expressed as estimated average annual percentage change (AAPC) and annual percent change (APC) with relative 95% confidence interval (95% CI), stratified by level of urbanization, sex, age, and race.

**Results:**

Between 1999 and 2019, 30,540 US subjects aged ≥ 65 years old (11,986 men and 18,554 women) equating to 77.8 deaths per 100,000 or 27.9 deaths per week, had iron deficiency anemia listed as a cause of death. The AAMR remained stable from 1999 to 2013 [APC: -0.3, (95%CI: -0.9 to 0.1, *p* = 0.11)] and then sharply increased from 2013 to 2019 [APC: +9.7% (95%CI: 7.8 to 11.6), *p* < 0.0001) without differences in sex, race, ethnicity or level of urbanization. The higher AAMRs were clustered in the Midwest [4.29 per 100,000 (95% CI: 4.20 to 4.38)] and in the South [3.35 per 100,000, 95% CI: 3.28 to 3.35)].

**Conclusions:**

Over the last two decades the iron deficiency anemia-related mortality trends increased among US older subjects, without differences by sex, race, ethnicity or urbanicity.

**Supplementary Information:**

The online version contains supplementary material available at 10.1007/s40520-025-02982-0.

## Introduction

Iron deficiency anemia in older subjects represents a common finding and a public health problem associated with morbidity and mortality [1]. Indeed, iron deficiency anemia negatively impacts everyday activity and is associated with poor quality of life and physical deterioration. Additionally, iron deficiency anemia is linked to higher rates of cardiovascular disease [2, 3], cancer [4], cognitive impairment [5] and increased risk of falls and/or fractures [6, 7]. Previous investigations, based on the National Health and Nutrition Examination Survey (NHANES) registry, estimated that about 20% of US older adults have an iron deficiency anemia with an expected rise with population aging [8–11]. Unfortunately, this reversible clinical condition often remains under-diagnosed in older subjects [8]. Moreover, data are lacking as regards the iron-deficiency anemia-related mortality trends in US subjects over 65 years of age. To fill this gap, we analyzed data on the primary cause of death reported in death certificates from the Centers for Disease Control and Prevention’s (CDC) Wide-ranging ONline Data for Epidemiologic Research (WONDER) dataset [12] to assess trends in US mortality related to iron-deficiency anemia over the past 21 years, and to determine possible differences in trends according to sex, race, ethnicity and census region.

## Study design and methods

### Data source

Data were retrieved through the publicly available CDC WONDER [12] dataset, which provides information from death certificates of all US residents according to the International Classification of Diseases, Tenth Revision (ICD-10). The database also includes demographic, geographical, and diagnostic data such as age, sex, race, ethnicity, and urbanization category. Iron-deficiency anemia-related deaths were ascertained when the ICD-10 codes D50 was listed in any position on the death certificate, defined as the disease or event that started the chain of events that led to death. Moreover, a sensitivity analysis considering iron deficiency anemia as the primary cause of death in the death certificate, was also performed. This selection strategy was in accordance with previous investigations exploring the epidemiological aspects of iron-deficiency anemia [13–15]. Although ICD-10 code was implemented in billing and care of patients at the end of 2015, the World Health Organization (WHO) authorized the publication of ICD-10 in 1999. This was implemented for mortality coding and classification of cause-of-death on death certificates in the US beginning in 1999 and spanning the entire study period [16].

For sex, race- and ethnicity-specific estimates, we used annual national population totals for sex, age group, race, and Latinx/Hispanic ethnicity obtained from the US Census Bureau [17]. The stratification between urban and rural counties was performed in accordance with the 2013 National Center for Health Statistics Urban-Rural Classification Scheme. Furthermore, mortality trends were analyzed by US census region (Northeast; Midwest; South and West). The study did not require institutional review board approval since the analysis was based on de-identified and publicly available data.

### Data extraction

Data extraction and validation were performed separately by two independent investigators (MZ, GZ). We abstracted the number of patients with iron deficiency anemia at the time of death, the population size from 1999 to 2019 as well as data on age, sex, race, ethnicity and census region in older US subjects, defined as those aged ≥ 65 years. “Ethnicity” was defined as Latinx/Hispanic and non-Latinx/Hispanic (NH), while “race” was categorized as Whites and Blacks. We did not perform specific sub-analyses on American Indians/Alaska natives, due to several missing data points; however, mortality estimates for this ethnic group were included in the general analysis as well as in those stratified by gender, age and urbanicity.

### Statistical analyses

Iron deficiency anemia age-adjusted mortality rates (AAMR) per 100,000 people with the relative 95% confidence interval (CI), were calculated by standardizing the related deaths using the annual national population totals from the US Census Bureau and the 2000 US standard population [17]. To calculate nationwide annual trends in iron deficiency anemia related mortality, we assessed the average percent change (APC) as well as average annual percent change (AAPC) and relative 95% confidence intervals (CIs). Considering that the abstracted data contains 21 time points, we identified a maximum of four inflection points across the study period, as currently suggested by the guidelines [18]. Statistical analyses were performed using Joinpoint regression (Joinpoint, version 4.6.0.0, National Cancer Institute, USA). Joinpoint regression software determines the inflection points for each population of interest, and accordingly across the study period, time intervals of interest vary. A parallelism test was used to examine whether groups have similar or different trends. Specifically, a significant p value on this interaction test indicated that the two trends, in terms of APCC, were statistically significantly different from each other. Statistical significance was prespecified at *p* ≤ 0.05 for findings in the entire population.

## Results

### Overall population and iron deficiency anemia at the time of death

**Fig. 1:**
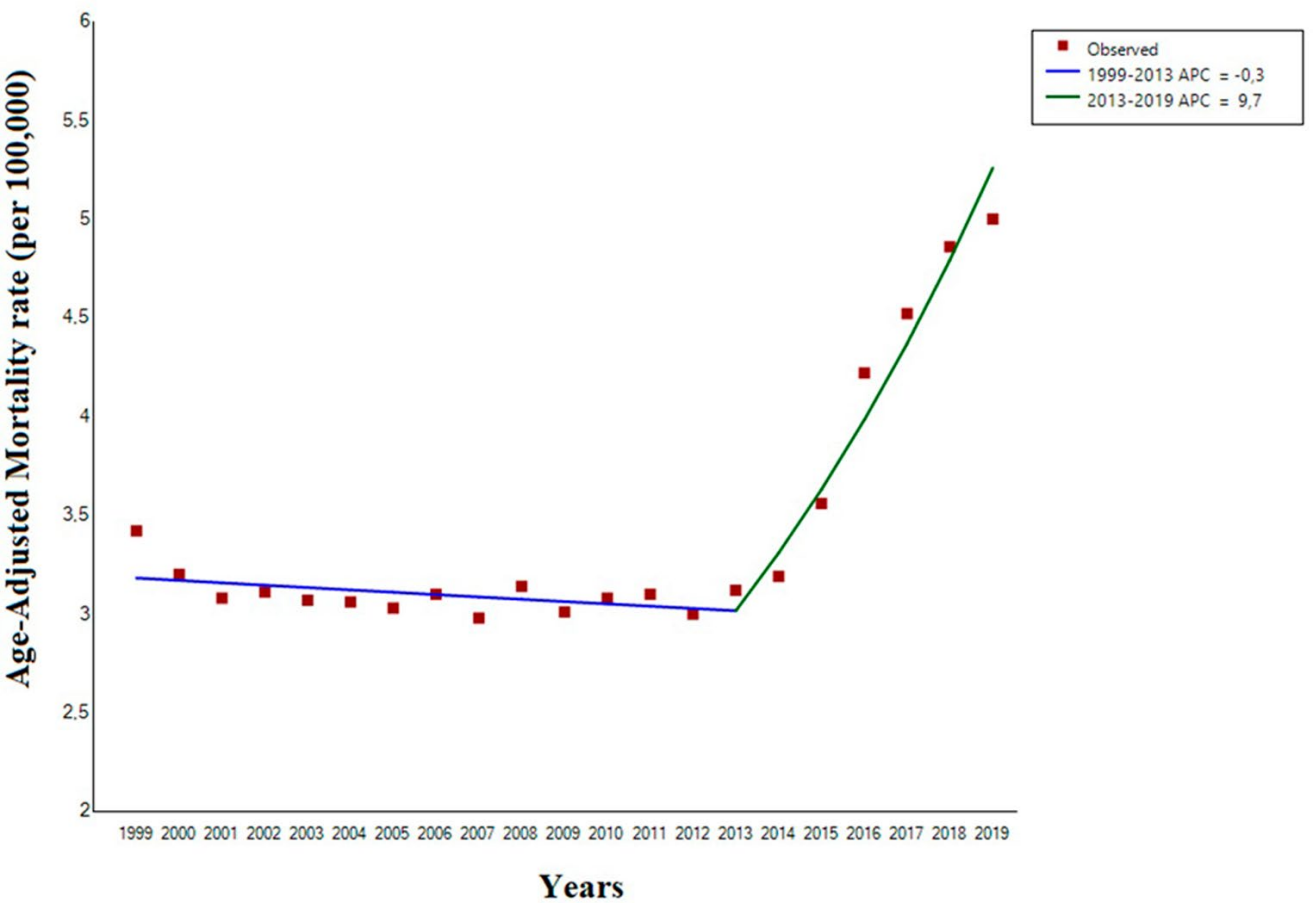
Trends in age-adjusted mortality rates related to iron deficiency anemia mortality in United States subjects aged ≥ 65 years old, 1999 to 2019

In the period from 1999 to 2019, there were 53.4 million deaths in the US. Among these, 30,540 older subjects (11,986 men and 18,554 women) had iron deficiency anemia listed as a cause of death equating to 77.8 deaths per 100,000 or 27.9 deaths per week. During this period, the AAMR for iron deficiency anemia increased from 3.42 (95%CI: 3.22 to 3.62) per 100,000 population in 1999 to 5.00 (95%CI: 4.81 to 5.19) population in 2019 [AAPC: +2.5% (95%CI: 2.0 to 3.1), *p* < 0.001] (Table 1). Notably, the AAMR remained stable from 1999 to 2013 [APC: -0.3, (95%CI: -0.9 to 0.1, p:0.11)], and then sharply increased from 2013 to 2019 [APC: +9.7% (95%CI: 7.8 to 11.6), *p* < 0.0001) (Fig. 1). The mortality rate, in terms of proportional mortality and AAMR, increased with population aging (Supplementary file 1).

**Table 1:** Age-Adjusted mortality rate trend in united States subjects aged 65 years or more with iron deficiency anemia, 1999–2019, stratified by sex and race/ethnicity. AAMR: Age-adjusted mortality rate, expressed as deaths per 100.000 population. AAPC: average annual percent change; APC: annual percent change

	AAMR 1999 (95%CI)	AAMR 2019 (95%CI)	AAPC; (95% CI), *p*	Number of Joinpoints	Period 1 [years] APC; (95%CI), *p*	APC - period 2 [years] APC; (95%CI), *p*	*p* for parallelism
Overall	3.42 (3.22 to 3.62)	5.00 (4.81 to 5.19)	+ 2.5; (2.0 to 3.1), *p* < 0.001	1	[1999–2013] -0.3; (-0.9 to 0.1), *p* = 0.11	[2013–2019] + 9.7; (7.8 to 11.6) *p* < 0.001	-
Male	3.63 (3.28 to 3.98)	5.50 (5.18 to 5.82)	+ 2.9; (1.9 to 3.9), *p* < 0.001	1	[1999–2013] -0.3; (-1.1 to 0.5), *p* = 0.43	[2013–2019] + 10.8; (7.7 top 14.0) *P* < 0.001	< 0.001
Female	3.32 (3.08 to 3.56)	4.67 (4.43 to 4.91)	+ 2.3;(1.6 to 3.1), *p* < 0.001	1	[1999–2013] -0.3; (-1.0 to 0.21), *p* = 0.21	[2013–2019] + 8.9; (6.5 to 11.4) *p* < 0.001	
**Race**							
White	3.46 (3.25 to 3.66)	5.12 (4.91 to 5.33)	+ 2.5; (1.9 to 3.1), *p* < 0.001	1	[1999–2012] -0.6; (-1.2 to -0.1), *p* = 0.04	[2012–2019] + 8.6; (6.9 to 10.2), *p* < 0.001	White vs. Blacks: *p* = 0.61 Blacks vs. Asian/Pacific Islanders: *p* = 0.73 White vs. Asian/Pacific Islanders: *p* = 0.81
Black or African American	3.93 (3.18 to 4.69)	5.45 (4.77 to 6.13)	+ 1.9; (0.3 to 3.5), *p* = 0.02	1	[1999–2013] -1.7; (-3.0 to -0.4), *p* = 0.01	[2013–2019] + 1.9; (0.3 to 3.5), *p* = 0.02	
Asian/Pacific Islanders	1.1 (1.0 to 1.2)	2.10 (1.57 to 2.76)	+ 2.7; (1.3 to 4.0), *p* < 0.001	0	-	-	
**Ethnicity**							
Latinx/Hispanic	3.07 (2.20–4.16)	3.69 (3.10–4.28)	+ 1.7; (-2.0 to 5.5), *p* = 0.38	1	[1999–2012] -1.9; (-5.4. to 1.7), *p* = 0.27	[2012 to 2019] + 8.7; (-0.9 to 19.2), *p* = 0.07	-

### Iron deficiency anemia as the underlying cause of death

Iron deficiency anemia was listed as the underlying cause of death in 3,861 US subjects aged ≥ 65 years old (1,331 males and 2,530 females). Also in this case, the AAMR significantly increased from 0.35 (95% CI: 0.31 to 0.38) per 100,000 population in 1999 to 0.56 (95% CI: 0.52 to 0.60) per 100,000 population in 2019 [AAPC: +1.8% (95% CI: 1.5 to 2.1). *p* < 0.001].

### Sex

**Fig. 2:**
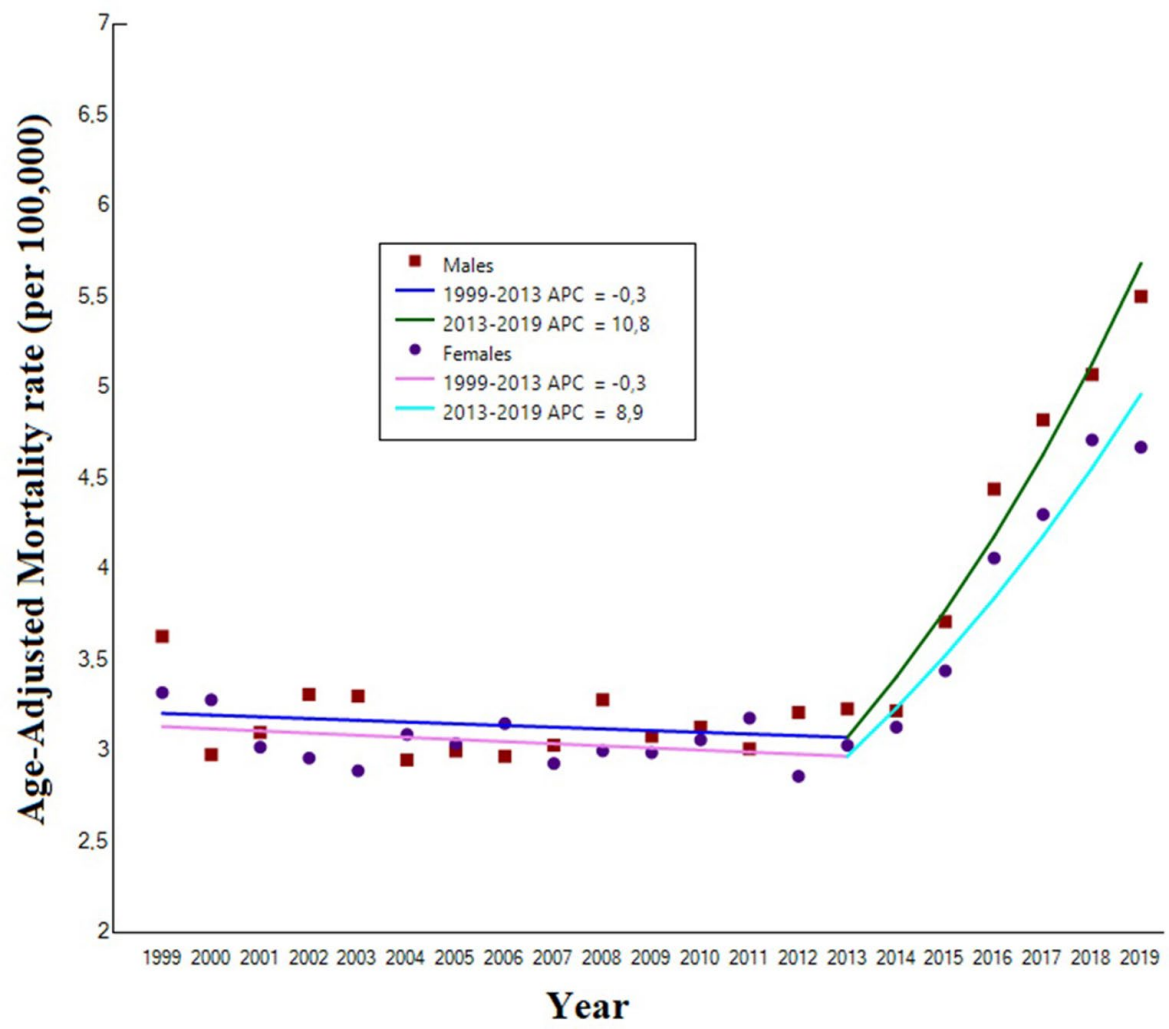
Trends in age-adjusted mortality rates related to iron deficiency anemia mortality in United States subjects aged ≥ 65 years old, stratified by sex, 1999 to 2019

US men and women experienced a similar AAMR increase over the entire study period (p for parallelism 0.62). In men the AAMR increased from 3.63 (95% CI: 3.28 to 3.98) per 100,000 in 1999 to 5.50 (95% CI: 5.18 to 5.82) per 100,000 in 2019 [AAPC: +2.9% (95% CI 1.9 to 3.9), *p* < 0.001]. More specifically, the mortality rate remained stable from 1999 to 2013 [APC: -0.3, (95% CI -1.1 to 0.5), *p* = 0.43) and then increased from 2013 to 2019 (APC: +10.8% (95% CI: 7.7 to 14.0), *p* < 0.001]. Similarly, in women the AAMR increased from 3.32 (95% CI: 3.08 to 3.56) per 100,000 in 1999 to 4.67 (95%CI: 4.43 to 4.9) per 100,000 in 2019. Also in this group, the mortality rate remained stable from 1999 to 2013 [APC: -0.3%, (95%CI: -1.0 to 0.2), *p* = 0.21] and then increased from 2013 to 2019 [APC: +8.9% (95%CI: 6.5 to 11.4). *p* < 0.0001] (Table 1; Fig. 2). A significant increasing trend was also observed considering iron-deficiency anemia as the underlying cause of death in both sexes [AAPC: +1.6% (95% CI: 1.3 to 1.9), *p* < 0.001 and AAPC: +1.9% (95% CI: 1.7 to 2.2), *p* < 0.001, in males and females, respectively.

### Race and ethnicity

In White individuals, the AAMR for iron deficiency anemia rose from 3.46 (95%CI: 3.25 to 3.66) per 100,000 in 1999 to 5.12 per 100,000 (95%CI: 4.91 to 5.33) in 2019 [AAPC: +2.5% (95%CI: 1.9 to 3.1), *p* < 0.001)]. Specifically, the AAMR slightly decreased from 1999 to 2012 [APC: -0.6%, (95%CI: -1.2 to -0.1, *p* = 0.04)] and then increased from 2012 to 2019 [APC: +8.6% (95% CI: 6.9 to 10.2), *p* < 0.001) (Table 1). Similarly, in (Non-Latinx/Hispanic) Black individuals, the AAMR rose from 3.93 (95%CI: 3.18 to 4.69) in 1999 to 5.45 (95%CI: 4.77 to 6.13) in 2019 [AAPC: +1.9% (95%CI: 0.3 to 3.5), *p* = 0.02)]. Notably, the relative AAMR decreased from 1999 to 2013 [APC: -1.7% (95%CI: -3.0 to -0.4), *p* = 0.01] and then significantly increased from 2013 to 2019 [APC: +1.9% (95%CI: 0.3 to 3.5), *p* = 0.02] (Table 1). A linear increase (no joinpoints detected) was observed among Asian/Pacific Islanders [AAPC: +2.7% (95%CI: 1.3 to 4.0), *p* < 0.001]. Indeed, in this group, the AAMR rose from 1.1 (95%CI: 1.0 to 1.2) per 100,000 in 1999 to 2.10 (95%CI: 1.54 to 2.7) per 100,000 in 2019. Finally, in Latinx/Hispanic individuals, the AAMR remained stable over the entire study period [AAPC + 1.7% (95%CI -2.0 to 5.5), *p* = 0.38), despite an increasing trend from 2012 to 2019 [APC: +8.7%, (95%CI: -0.9 to 19.2), *p* = 0.07) which did not reach statistical significance.

### Contributing causes of death

The main contributing causes of death (representing ≥ 3% of deaths) related to iron deficiency anemia, are reported in Supplementary File 2. Among these, the first three were atherosclerotic heart disease (n: 1928; 6.3%), gastrointestinal hemorrhage (n:1427; 4.6%), and colon malignancies (n:1262; 4.1%) (Supplementary file 2).

### Geographical patterns

**Fig. 3:**
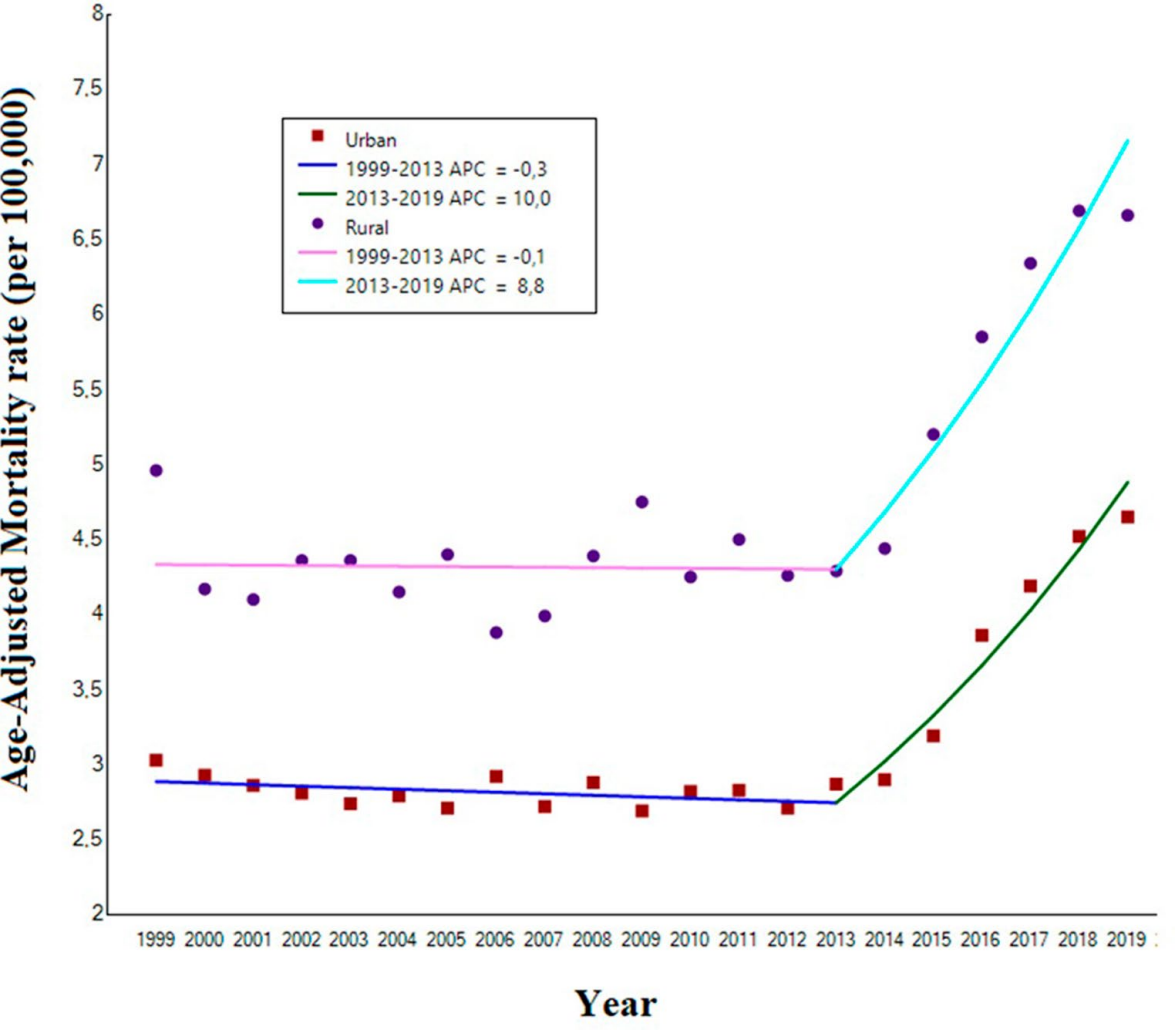
Trends in age-adjusted mortality rates related to iron deficiency anemia mortality in United States subjects aged ≥ 65 years old, stratified by urbanicity, 1999 to 2019

Between 1999 and 2019, iron deficiency anemia-related mortality increased both in urban and rural areas, without significant differences [AAPC: +2.7% (95%CI: 2.0 to 3.3), *p* < 0.001 and AAPC: +2.5% (95%CI: 1.4 to 3.7), *p* < 0.001 for urban and rural areas, respectively] (Table 2; Fig. 3). The higher AAMRs were clustered in the Midwest [4.29 per 100,000 (95%CI: 4.20 to 4.38)] and in the South [3.35 per 100,000, 95%CI: 3.28 to 3.35)] (Supplementary file 3).

**Table 2:** Age-Adjusted mortality rate trend in united States subjects aged 65 years or more with iron deficiency anemia, 1999–2019, stratified by level of urbanization. AAMR: Age-adjusted mortality rate, expressed as deaths per 100.000 population. AAPC: average annual percent change; APC: annual percent change

	AAMR 1999 (95% CI)	AAMR 2019 (95% CI)	AAPC; (95% CI), *p*	Number of Joinpoints	Period 1 [years] APC; (95% CI), *p*	APC - period 2 [years] APC; (95% CI), *p*	*P*
**County-level urbanization**							
Urban	3.03 (2.82 to 3.23)	4.65 (4.45–4.85)	+ 2.7; (2.0 to 3.3), *p* < 0.001	1	[1999–2013] -0.3; (-0.96 to 0.2), *p* = 0.16	[2013 to 2019] + 10.0; (8.0 to 12.1), *p* < 0.001	0.78
Rural	4.96 (4.43–5.49)	6.66 (6.11 to 7.20)	+ 2.5; (1.4 to 3.7), *p* < 0.001	1	[1999–2013] -0.1; (-1.0 to 0.9) *p* = 0.90	[2013 to 2019] + 8.5; (8.8 to 12.7), *p* < 0.001	

## Discussion

Our study highlights a worrying trend in iron deficiency anemia-related mortality among older adults. After a plateau from 1999 to 2013, the AAMR for iron deficiency anemia significantly increased without sex, race, or ethnic differences. However, a greater increase was observed in the Midwest and Southern regions. This increasing mortality trend is consistent with previous nationwide epidemiological analyses demonstrating an increasing prevalence of iron deficiency anemia in the US [19–21]. The observed trends are likely attributable to a confluence of different risk factors. Doubtless, the increasing prevalence represented a major contributing factor [19–21]. Indeed, previous investigations have demonstrated that the prevalence of anemia increases parallel to aging and life expectancy [8, 21]. In this regard, it is possible that, due to the continuous increase in life expectancy in the US population, a growing number of people have reached an advanced age, thus developed iron-deficiency anemia and increasing the related mortality rate [22, 23]. Furthermore, a lower dietary iron intake as well as a decreasing iron concentration in US food products have been reported in the last decade [11]. Additionally, a greater incidence and prevalence of several chronic conditions, such as chronic inflammatory disease, chronic kidney disease, and cancers have probably contributed to the observed trends [24–29]. On the other hand, some typical conditions of older subjects, including vitamin deficiencies, malabsorption, and partial/complete edentulous state might have participated in the increase in mortality rate [30, 31]. Another contributing factor is probably represented by the increasing prevalence of depression in US older adults which represents an established risk factor for malnutrition due to its ability to decrease appetite and food intake [8–11]. Finally, the widespread use of non-steroidal anti-inflammatory drugs, antithrombotic drugs, anticoagulants, and proton pump inhibitors may trigger blood loss and therefore iron deficiency anemia [32].

Results regarding sex, ethno-racial, and regional sub-groups deserve particular attention. Indeed, we observed that the iron deficiency anemia AAMR increased in all sub-groups, suggesting that iron deficiency anemia is a diffuse and potentially reversible aging-associated condition largely unrecognized in daily clinical practice, despite the diagnosis being easy, unless the condition is masked by inflammatory conditions [33]. Moreover, the diagnosis of iron deficiency anemia is often delayed since, in the early stages, it is characterized only by iron deficiency and patients are often pauci- or asymptomatic; unfortunately, long-standing iron deficiency anemia may be challenging to treat.

Doubtless, the unequal distribution of mentioned risk factors, especially among disadvantaged groups, remains a major determinant of different trends. In the present analysis, Whites experienced the greatest rise in their iron deficiency anemia-related mortality trends. This result may be explained, at least in part, by the higher prevalence of depression among these subjects as well as by the higher occupancy rate of nursing homes [34]. In our analysis the major inflection point regarding the iron deficiency anemia AAMR was observed in the years 2012–2013. The reasons for this are probably multiple. Indeed, in those years, some international and US scientific societies released specific guidelines for the identification and treatment of some chronic diseases associated to iron anemia deficiency, including CKD and cancer [35–37]. It is plausible that the reported increasing mortality rate may be, at least in part, related to better recognition of some previously ‘unexplained’ deaths. Moreover, colorectal cancer screening increased in the US from 2012 [38]. Therefore, an increasing number of patients were tested for iron deficiency anemia, since colorectal cancer is the most frequent cause for blood loss in older subjects. Our analysis showed that the Midwest and West were the census regions with the highest AAMR. Several contributing factors may explain such trends such as the increasing poverty among older subjects in these areas, including aging- associated decline in hemoglobin levels nutritional deficiencies, decreased erythropoietin production, and chronic disease. However, the CDC WONDER dataset, being a mortality registry, did not allow us to adjust our findings for these potential confounders [34]. Although our results provide an updated analysis of US iron deficiency anemia-related mortality, further research is warranted to understand which of the contributing factors may be the primary driver of these trends.

### Limitations

Our analysis is based on a large nationwide administrative dataset and the findings should be interpreted within the limitations of the data. First, the retrospective design of the investigation, with all inherited bias, and/or the inaccuracy of death certificate information may have partially biased our findings. Second, we cannot analyze the incidence or prevalence of iron deficiency anemia, since these data were not provided by the CDC WONDER dataset. Third, our results may underestimate the real prevalence of iron-deficiency anemia at the time of death, since we did not capture the cases that remain undiagnosed. Fourth, we cannot provide specific sub-analyses regarding American Indians/Alaska Natives due to several missing data points. Additionally, miscoding of iron deficiency anemia as the underlying or contributing cause of death may have impacted the accuracy of our results. However, previous investigations have validated the use of the ICD-10 code D50, reporting that its use for the estimation of prevalence or mortality of iron-deficiency anemia was associated with a high positive predictive value. Finally, since the CDC WONDER database is a mortality dataset, it does not include any data regarding baseline comorbidities, symptoms, laboratory findings, and therapeutic strategies, thus making any sub-analysis impossible.

## Conclusions

Iron deficiency anemia is recognized as a major public health issue, especially in older subjects, and is associated with substantial morbidity and mortality. After a plateau between 1999 and 2013, the iron deficiency anemia-related mortality rate increased significantly in the US in older subjects, without significant differences in sex, race, ethnicity, or urban location. Further research might be carried out to explain observed trends. Intensification of public health efforts is needed, aimed at early identification and treatment of older patients with iron deficiency anemia, and to reduce the related mortality trends.

## Electronic supplementary material

Below is the link to the electronic supplementary material.

Supplementary Material 1

Supplementary Material 2

Supplementary Material 3

## Data Availability

No datasets were generated or analysed during the current study.
