# Supplementary material for: Iron deficiency anemia-related mortality trends in US older subjects, 1999 to 2019
Source: Aging Clin Exp Res. 2025 Mar 22;37(1):99. doi: 10.1007/s40520-025-02982-0 (PMC11928430; doi:10.1007/s40520-025-02982-0)
Supplement: Supplementary file 1 — Supplementary Material 1 [file 40520_2025_2982_MOESM1_ESM.docx]

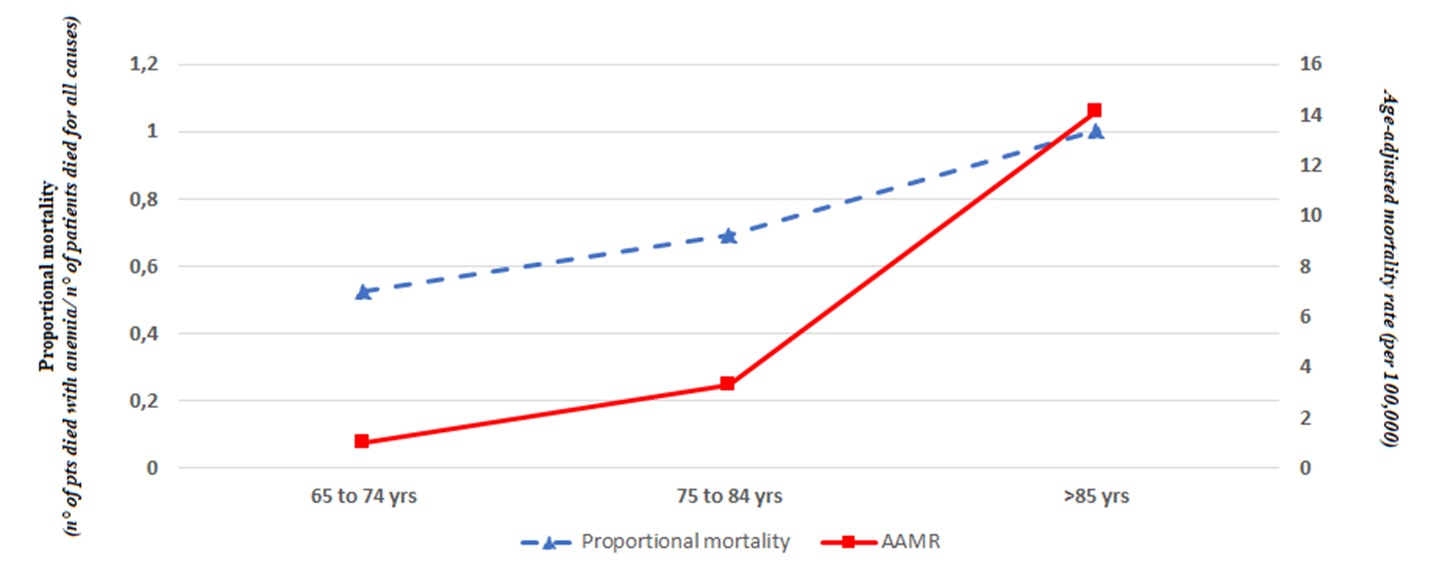


Proportional mortality and age-adjusted mortality rate in patients with iron-deficiency anemia at the time of death in US, 1999&#x2013;2019. AAMR: Age-adjusted mortality rate.
