# Supplementary material for: Iron deficiency anemia-related mortality trends in US older subjects, 1999 to 2019
Source: Aging Clin Exp Res. 2025 Mar 22;37(1):99. doi: 10.1007/s40520-025-02982-0 (PMC11928430; doi:10.1007/s40520-025-02982-0)
Supplement: Supplementary file 2 — Supplementary Material 2 [file 40520_2025_2982_MOESM2_ESM.docx]

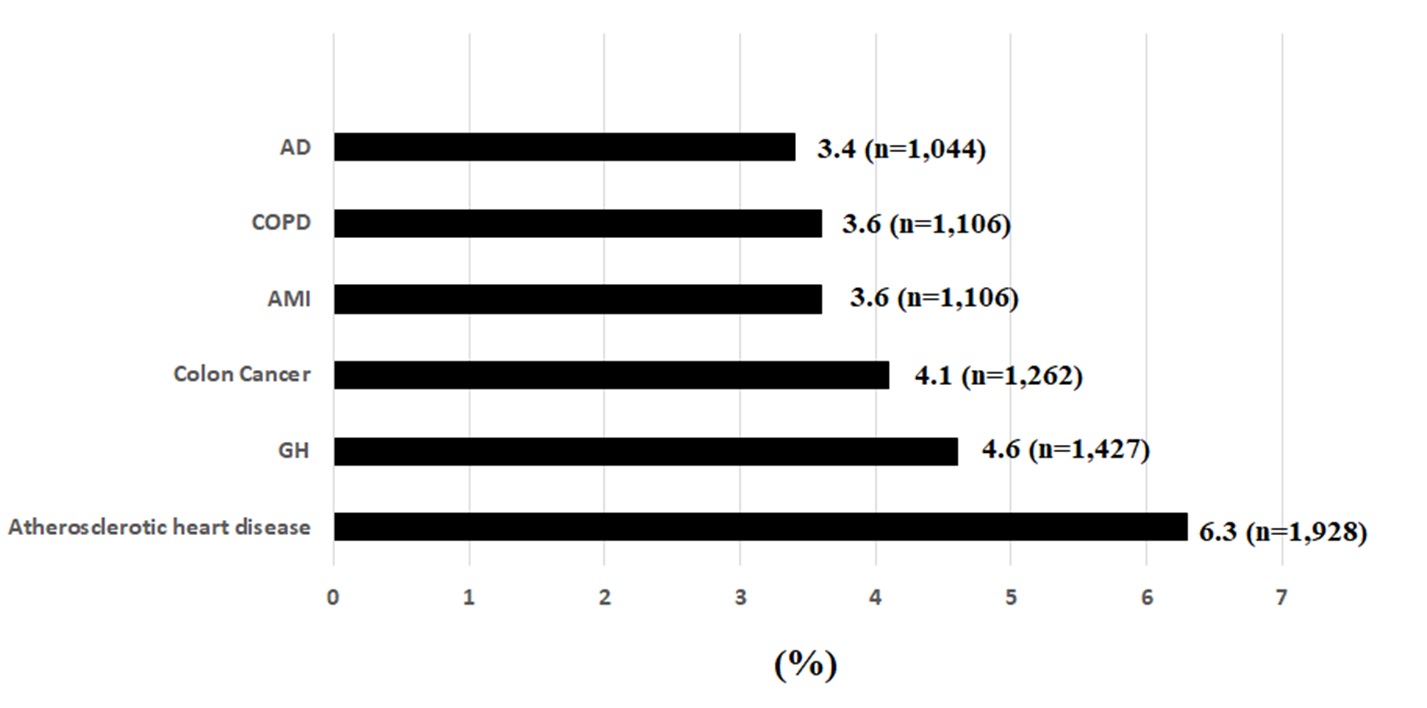


Contributing causes of death in US subjects aged&#x2009;&#x2265;&#x2009;65 years old died with iron deficiency anemia. AD: Alzheimer&#x2019;s disease; COPD: Chronic obstructive pulmonary disease; AMI: Acute myocardial infarction; GH: Gastrointestinal hemorrhage.
