# Supplementary material for: Iron deficiency anemia-related mortality trends in US older subjects, 1999 to 2019
Source: Aging Clin Exp Res. 2025 Mar 22;37(1):99. doi: 10.1007/s40520-025-02982-0 (PMC11928430; doi:10.1007/s40520-025-02982-0)
Supplement: Supplementary file 3 — Supplementary Material 3 [file 40520_2025_2982_MOESM3_ESM.docx]

| **Census region** | **n** | **AAMR (per 100,000 people)** | **95% CI** |
| --- | --- | --- | --- |
| **Northwest** | 5,294 | 2.89 | 2.81 to 2.97 |
| **Midwest** | 8,716 | 4.29 | 4.20 to 4.38 |
| **South** | 10,476 | 3.35 | 3.28 to 3.35 |
| **West** | 6,054 | 3.27 | 3.19 to 3.35 |

**Table 3**. Age-Adjusted Mortality rate related iron deficiency anemia in in US subjects, 1999-2019 aged 65 years old or more, according to the census region. AAMR: Age-adjusted mortality rate; CI: Confidence interval.
